# Expression of Th1/2/17 Cytokines in CML with or without Pulmonary Bacterial and Fungal Coinfection

**DOI:** 10.1155/2023/6318548

**Published:** 2023-04-18

**Authors:** Xin Guan, Chaoran Zhang, Peng Hu, Zefeng Yang, Jinping Zhang, Yunlian Zou, Yan Wen, Huiyuan Li, Tonghua Yang, Renbin Zhao, Zengzheng Li

**Affiliations:** ^1^Department of Hematology, The First People's Hospital of Yunnan Province, Affiliated Hospital of Kunming University of Science and Technology, Kunming, China; ^2^Yunnan Blood Disease Clinical Medical Center, The First People's Hospital of Yunnan Province, Kunming, China; ^3^Yunnan Blood Disease Hospital, The First People's Hospital of Yunnan Province, Kunming, China; ^4^National Key Clinical Specialty of Hematology, The First People's Hospital of Yunnan Province, Kunming, China; ^5^Yunnan Clinical Medical Research Center for Hematological Diseases, Kunming, China

## Abstract

**Background:**

Tyrosine kinase inhibitors (TKIs) are the standard therapy for patients with chronic myeloid leukemia (CML). While their use greatly increases patient survival rates and can lead to normal life expectancy, bacterial infections in the lungs continue to play a significant role in determining patient outcomes.

**Methods:**

In this study, the medical records of 272 CML and 53 healthy adults were analyzed. Information on age, sex, body temperature, procalcitonin (PCT), C-reactive protein (CRP), and cytokine levels were collected from patients. Since the data belonged to a nonstate distribution, we used the Mann–Whitney *U* test to examine differences between groups. Cut-off values were analyzed by receiver operating characteristic (ROC) curves.

**Results:**

No significant differences in the Th1/2/17 levels were observed in relation to TKI treatment. Further analysis showed that the levels of the interleukins IL-2, IL-4, IL-5, IL-6, IL-8, IL-10, IL-22, IL-12p70, IL-17A, IL-17F, and IL-1*β*, interferon (IFN-*γ*), and tumor necrosis factors (TNF *α* and *β*) were higher in patients with pulmonary bacterial infections compared with uninfected patients. IL-6, IL-8, and IL-10 levels in CML patients with bacterial and fungal coinfection were higher than those in patients without infection. The areas under the ROC curves (AUCs) were found to be 0.73 for IL-5, 0.84 for IL-6, 0.82 for IL-8, 0,71 for IL-10, and 0.84 for TNF-*α*. AUC values were higher for patients with pulmonary bacterial infection, especially IL-6 (AUC = 0.84, cut-off = 13.78 pg/ml) and IL-8 (AUC = 0.82, cut-off = 14.35 pg/ml), which were significantly better than those for CRP (AUC = 0.80, cut-off = 6.18 mg/l), PCT (AUC = 0.71, cut-off = 0.25 ng/ml), and body temperature (AUC = 0.68, cut-off = 36.8°C). In addition, according to the cut-off values, we found that 83.33% of patients with pulmonary bacterial infections had IL-6 ≥ 13.78 pg/ml, while when IL-6, IL-8, and IL-10 levels simultaneously exceeded the cut-off values, the probability of pulmonary bacterial infection was 93.55%.

**Conclusions:**

TKI treatment did not appear to affect cytokine expression in CML patients. However, CML patients with pulmonary bacterial infection had significantly higher levels of Th1/2/17 cytokines. In particular, abnormally elevated IL-6, IL-8, and IL-10 levels were associated with a pulmonary bacterial infection in patients with CML.

## 1. Introduction

Chronic myeloid leukemia (CML) is a hematologic malignancy characterized by uncontrolled growth of myeloid cells in the bone marrow. The introduction of tyrosine kinase inhibitors (TKIs) has enhanced life expectancy in many patients with CML [1–3]. However, minor clinical symptoms can be easily ignored by doctors when patients on well-established treatment regimens visit the outpatient clinic. This can lead to untreated conditions, further exacerbating infections. The presence of bacterial lung infections can influence the patient's quality of life and increase the cost of treatment, especially if a mild infection becomes more severe [4, 5].

Biomarkers play a significant role in diagnosing diseases that cannot be detected with the naked eye or by other testing. The most commonly used clinical biomarkers for infection are procalcitonin (PCT) and C-reactive protein (CRP) [6]. CRP plays a key role in recognizing acute phase reactants released by infection, tissue damage, and inflammation mediated by the cytokine IL-6 [7]. PCT is a precursor of the hormone calcitonin and is synthesized by C cells in the thyroid and neuroendocrine cells in the intestine and lungs. The levels of PCT are significantly increased in patients with severe septic shock or sepsis [8]. However, many studies on predicting respiratory system infection have found that the sensitivity of both these markers to local infection, especially lung infection, is lower than that of other markers, such as cytokines [5, 9, 10]. While tests such as sputum culture have always been important in the detection and diagnosis of respiratory system infections, these also have limitations, especially the extended time required for sputum culture, as well as its proneness to secondary infection and relatively low sensitivity [11]. Blood cultures are less able to detect pulmonary infection and are generally not considered in the absence of typical features. Computed tomography (CT) and chest X-rays are also used to confirm the diagnosis. However, CT is not usually conducted in the absence of obvious symptoms of infection, which can lead to delayed diagnosis and worsening of the infection.

Recent research has focused on the predictive ability of elevated cytokine levels in infections in various populations. Previous studies have reported that COVID-19 infection in elderly vulnerable patients to a cytokine storm, characterized by severe systemic elevation of several proinflammatory cytokines. Similarly, some studies have also observed raised cytokine levels in patients (both adults and children) with hematological malignancies, community-acquired pneumonia, and non-Hodgkin's lymphoma, and reported that cytokine levels have greater diagnostic sensitivity than both PCT and CRP [12–15]. Although previous studies have demonstrated that cytokines can predict infection in patients with hematological malignancies, these conditions differ markedly in both pathogenesis and immune characteristics, and cytokine production may also be affected by drug therapy. The current study aimed to investigate the usefulness of cytokine levels in CML patients with and without treatment, specifically, patients with CML who were either receiving TKI therapy or not.

## 2. Materials and Methods

### 2.1. Patients and Data Collection

In this study, the medical records of 272 patients with CML and 53 healthy adult patients between December 2017 and May 2022 were obtained from the Hematology Department of Yunnan First People's Hospital. Each patient provided informed consent in written form. Of these, 65 patients had pulmonary bacterial infection, 172 had no infection, 19 were infected with hepatitis B virus (HBV), and 16 had simultaneous bacterial and fungal infection. The body temperatures and CRP, PCT, and Th1/2/17 cytokine levels were initially recorded. The diagnostic criteria for pulmonary infection [16–18] were suspected or confirmed bacterial lung infection, irrespective of the presence or absence of bacteria or fungi in respiratory specimens, determined by laboratory guidelines and clinical presentation, as well as new-onset or worsening cough, hypoxia, shortness of breath, or dyspnea. Additional auxiliary tests included X-rays of the chest to confirm sputum culture and inflammatory changes for the detection of bacterial/fungal infection. Chest CT scans were performed to confirm bacterial/fungal infection in patients that were suspected of having the pulmonary infection. The exclusion criteria included patients with trauma, determined by reviewing the description of the patient's physical condition, and those with autoimmune diseases such as systemic lupus erythematosus, rheumatoid arthritis, systemic vasculitis, scleroderma, multiple encephalomyelitis, hyperthyroidism, Sjogren's syndrome, and connective tissue disease. Patients with suspected autoimmune diseases were also excluded by reviewing the relevant clinical examinations, especially the determination of the presence of antinuclear antibodies. Furthermore, only patients with CML who received TKI treatment were included, and those who received other treatments such as bone marrow transplantation were excluded. An additional criterion was that the included patients did not receive anti-infection treatment. The study was approved by the First People's Hospital of Yunnan Province Ethical Committee were strictly followed in the study process (approval number, KHLL2022-KY075) and followed the requirements of the Helsinki Declaration, the Human Biomedical Research International Code of Ethics, World Health Organization (WHO), and the International Medical Science Organizations Council.

### 2.2. Measurement of Cytokines

The Aim Plex Cytokine Kit (QuantoBio, Tianjin, China) was used to measure the serum levels of cytokines, i.e., Th (1/2/17), interferon (IFN)-*γ*, IL (1*β*, 2, 4, 5, 6, 8, 10, 12p70, 17A, 17F, and 22), and tumor necrosis factors TNF-*α* and TNF-*β*, with a detection range of 1–2500 pg/ml using the NovoCyte D3000 flow cytometer (Agilent, Santa Clara, CA, USA).

### 2.3. Statistical Analysis

The normality of the data distribution was tested, and as the data were not normally distributed, the Mann–Whitney *U* test was used to compare differences between groups. Continuous data were presented as medians with IQR (interquartile range), and categorical data as frequencies and percentages. Receiver operating characteristic (ROC) curves were constructed, and the area under the curve (AUC) was used to evaluate the diagnostic efficacy of cytokines, CRP, and PCT. To identify the best cut-off value, the highest Uden index (sensitivity + specificity − 1) was used. GraphPad Prism 7 software was used to create distribution maps, and SPSS version 21.0 (IBM Corp., Armonk, NY, USA) was used for statistical evaluation. *P* values <0.05 (bilateral) were considered statistically significant.

## 3. Results

### 3.1. Patient Characteristics

**Figure 1:**
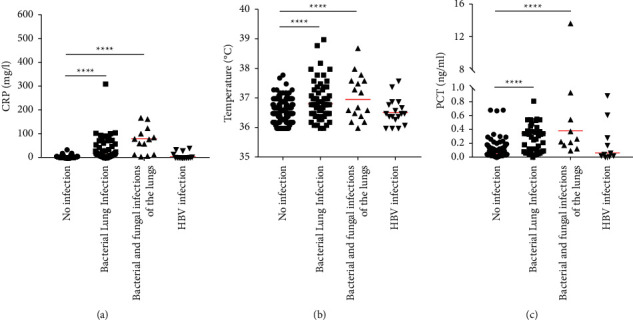
The Mann–Whitney *U* test was used to examine the differences between patients with respiratory bacterial infection, patients with respiratory bacterial and fungal infections, patients without bacterial infection, and patients with HBV infection. (a) CRP, (b) body temperature, and (c) PCT. ^*∗∗∗∗*^*P* < 0.0001, ns: no statistical difference.

A total of 325 patients were included in our cohort, of whom 53 were healthy patients, 272 were CML, 41.54% (113/272) were female, 58.46% (159/272) were male, and the median age was 43 (28–56) years. Of the CML patients, 63.24% had no detectable infection, while 23.89% were diagnosed with pulmonary bacterial infection, 6.99% with HBV infection, and 5.88% with pulmonary coinfections of bacteria and fungi (Table 1). The median body temperature of patients with no infection was 36.5 (36.2–36.8)°C, which was significantly lower than that in patients with bacterial infection (36.8, (36.5–37.2)°C) and bacterial and fungal coinfection (37.0, [36.4–37.6]°C). However, the temperature of patients with HBV infection (36.5, (36.3–36.8)°C) did not differ significantly from that of uninfected patients (Table 1 and Figure 1(b)). The median PCT of 0.05 (0.03–0.13) ng/ml in patients with no infection was significantly lower than that in patients with bacterial infection (0.35 (0.10–0.55) ng/ml) and bacterial and fungal coinfection (0.38 (0.20–3.12) ng/ml) but was not significantly different in patients infected with HBV (0.06 (0.03–0.37) ng/ml) (Table 1 and Figure 1(c)). The median CRP level of 2.75 (1.83–5.21) mg/l in uninfected patients was significantly lower than that in patients with bacterial infection (15.65 (8.17–54.85) mg/l) and bacterial and fungal coinfection (78.50 (13.75–113.50) mg/l) while the median CRP value of 2.70 (1.69–19.98) mg/l in patients with HBV infection was not significantly altered (Table 1 and Figure 1(a)).

### 3.2. Expression of Cytokines in Different Infections

**Figure 2:**
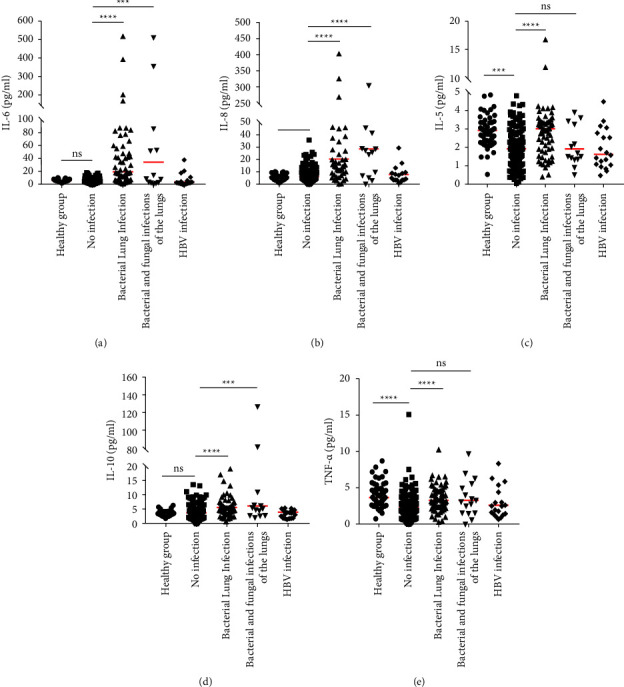
The Mann–Whitney *U* test was used to examine the differences between patients with respiratory bacterial infection, patients with respiratory bacterial and fungal infections, patients without bacterial infection, and patients with HBV infection. (a) IL-6, (b) IL-8, (c) IL-5, (d) IL-10, and (e) TNF-*α*. ^*∗∗∗*^*P* < 0.001, ^*∗∗∗∗*^*P* < 0.0001, ns: no statistical difference.

To determine the effects of TKI treatment on cytokine expression in CML patients, cytokine levels were assessed in uninfected CML patients. The results showed that there was no statistical difference in the expression of Th1/2/17 between patients treated with TKI and untreated patients (Table 2). Cytokine levels were then analyzed in the different patient groups, namely, the healthy group and CML patients with no infection, bacterial lung infection, bacterial and fungal coinfection, and HBV infection. We found that cytokine IL-4, IL-12P70, IL-1*β,* and IL-22 in noninfected CML patients is higher than that of healthy people (Table 3, Supplementary Figure 1(A)–1(C) and 1(I)), but IL-5, IFN-*γ*, TNF-*α*, TNF-*β,* and IL-17F is lower than the normal population (Table 3, Figures 2(c) and 2(e), Supplementary figure 1(E), 1(F) and 1(H)). Th1/2/17 cytokines in CML patients with a bacterial lung infection were higher than in those without infection (Table 3, Figures 2(a)–2(e), and Supplementary Figure 1(A)–1(I)). The levels of IL-6, IL-8, and IL-10 in CML patients with bacterial and fungal infections of the lung were significantly higher than those without infection (Table 3 and Figures 2(a), 2(b), and 2(d)). However, there were no statistical differences in cytokine levels between HBV-infected and uninfected CML patients, and there was also no significant difference between patients with bacterial infections only and those with both bacterial and fungal infections (Table 3).

### 3.3. Determination of Specific Indicators

**Figure 3:**
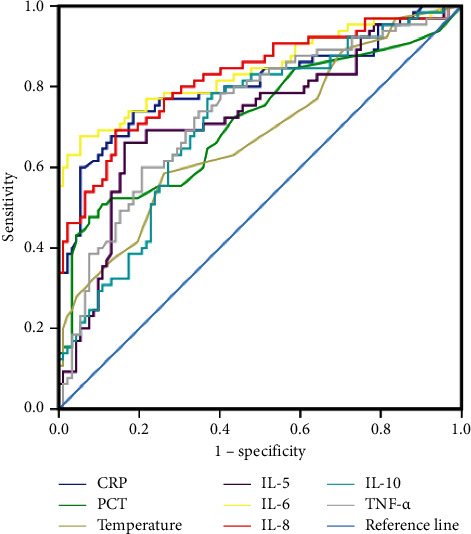
The ROC curves of cytokines, PCT, body temperature, and CRP in patients with respiratory tract bacterial infection were compared with those without respiratory tract infection.

The study cohort included relatively fewer cases of HBV infections and bacterial and fungal coinfections (*n* < 20, Table 1). Therefore, the levels of specific indicators were only compared between patients with and without bacterial infection in the lungs. The AUC and cut-off values on the ROC curves were higher for IL-6 (AUC = 0.84, cut-off = 13.78 pg/ml) and IL-8 (AUC = 0.82, cut-off = 14.35 pg/ml) compared with those for body temperature (AUC = 0.68, cut-off = 36.8 pg/ml), PCT (AUC = 0.71, cut-off = 0.25 ng/ml), and CRP (AUC = 0.80, cut-off = 6.18 mg/l). Moreover, the AUC values for IL-5 (AUC = 0.73, cut-off = 2.41 pg/ml), and TNF-*α* (AUC = 0.74, cut-off = 3.00 pg/ml) were higher than those for body temperature (AUC = 0.68, cut-off = 36.8 pg/ml) and PCT (AUC = 0.71, cut-off = 0.25 pg/ml) while the values for IL-10 (AUC = 0.71, cut-off = 4.01 pg/ml) was only higher than that for body temperature (AUC = 0.68, cut-off = 36.8°C) (Table 3 and Figure 3).

Furthermore, we calculated the probability of pulmonary bacterial infection in patients with values equal to or above the cut-off value. The results showed that values ≥cut-off value had the highest probability for IL-6 (83.33%), followed by PCT (75.61%) and IL-8 (67.16%) (Table 4). In addition, we analyzed the probability of pulmonary bacterial infection when the values for all three IL-6, IL-8, and IL-10 were equal to or above the cut-off values, finding that these patients had a 93.55% probability of pulmonary bacterial infection (Table 4).

## 4. Discussion

Tyrosine kinase inhibitors (TKIs) are the standard therapy for individuals with CML, resulting in significant improvement in survival rates and enabling many patients to live normal lives. However, despite the availability of effective treatments, bacterial infections in the lungs continue to influence patient outcomes. Abnormally high cytokine levels have been shown to predict infection in patients with hematological malignancies [19, 20]. Hence, the current study evaluated cytokine levels to predict infection associated with hematological disease. A previous study observed elevated levels of IL-6, IL-8, and IL-10 in newly diagnosed hematological malignancies accompanied by pulmonary bacterial infections [13]. Non-Hodgkin's lymphoma was analyzed separately, finding significantly raised levels of IL-6 (AUC = 0.92) in this disease [12]. The current study found that, apart from IL-6, changes in IL-8 levels exceeded those of PCT and CRP. In addition, patients with simultaneous increases in IL-6, IL-8, and IL-10 had a 93.55% probability of pulmonary bacterial infection.

IL-6 binds to its receptor on the cell membrane to form a complex (IL-6-IL-6R), activating the JAK-STAT pathway, and subsequently binds to glycoprotein 130 (gp 130) to form an IL-6-IL6R-gp130 complex [21, 22], leading to downstream signaling. Raised levels of IL-6 are one of the most common findings in infection, and IL-6 levels in the blood are effective for assessing the presence of infection [23]; IL-6 concentrations in sepsis patients can reach up to 1600 pg/mL [24], in contrast to normal adults where the levels are generally less than 7.8 pg/mL [25, 26]. Here, the optimal cut-off value was observed to be significantly higher than that of 7.8 pg/mL in CML without infection (Table 3 and Figure 2(a)). The expression level of IL-6 can also distinguish the severity of infection to some extent, as confirmed by previous findings [12]. The equally advantageous IL-8 signals primarily through the extracellular binding of CXCR1 (CXC-chemokine receptor 1) and CXCR2 (CXC-chemokine receptor 2). This signal recruits neutrophils to areas of inflammation to facilitate bacterial clearance and reduce inflammatory stimuli at the site of inflammation [27, 28].

Although aberrant expression of cytokines has been shown to have high clinical value in children and adults with hematological malignancies [5, 13, 29], differences between diseases should still be taken into account for a more complete understanding of the value of cytokines in diagnosis. In CML, it was found that Th1/Th2/Th17 cytokines were higher in patients with pulmonary bacterial infections than in patients without infection (*P* < 0.05, Table 3). However, in non-Hodgkin's lymphoma, IL-2, -4, -5, -12P70, -1*β*, -17A, -17F, -22, and TNF-*α* play critical roles in bacterial infections of the lungs [12]. This evidence suggests that cytokines are involved in many diseases and their expression profiles also differ in different diseases, although this does not affect the predominance of IL-6 and IL-8. Another difference from non-Hodgkin's lymphoma is that IL-5 (AUC = 0.73) and TNF-*α* (AUC = 0.74) in CML were also found to be useful in distinguishing bacterial infections in the lungs (Table 4). In addition, in this study, we found that there were no significant differences in the Th1/2/17 cytokine levels between uninfected patients who received TKI treatment and those who did not (Table 2). These results suggest that TKI administration may not affect the levels of IL-6 and IL-8. In this study, we also included CML patients with HBV infection, but no significant differences were observed in the Th1/2/17 cytokine levels compared with CML patients without HBV infection (Table 3).

This single-center study has some limitations. First, there were relatively few cases of fungal and bacterial coinfections and HBV infection, and more cases are needed to verify the results. Second, different forms of hematological malignancies should be investigated to comprehensively assess cytokine levels in hematological disorders. In addition, BAL culture and microbial next-generation sequencing (mNGS) detection are among the best methods for evaluating pulmonary infection. The use of fiberoptic bronchoscopy to obtain bronchoalveolar lavage fluid is defined as an invasive procedure, and is thus often refused by patients affected by traditional culture and fear of lung damage, especially in western China (regions with relatively backward economy and education). Therefore, very few patients have this test. mNGS has a wide detection range and allows accurate identification. However, the mNGS test cannot be performed in most hospitals in China, requiring outsourcing to a third-party company such as the BGI Genomics Institute, which is the most extensive in China. The economics of patients in developing countries such as China also require consideration and due to the restrictions of the regional health center policy, only some specialized tests in cooperation with third parties are available. Because of these issues, we cannot conduct mNGS on large numbers of samples. Of course, we believe that with the development of our country, mNGS testing will be popularized in most hospitals in the future. Thus, we support the use of mNGS to detect pathogenic microorganisms in patients with pulmonary infections to identify the responsible microorganisms and provide guidance for clinical treatment. However, the core challenge of mNGS is to distinguish pathogens from colonizing microorganisms, which also complicates the interpretation of the results and is not conducive to clinician interpretation [30, 31]. In contrast to mNGS, despite the presence of nonspecific cytokines, colonizing pathogens generally do not lead to cytokine disturbances [32–34]. In addition, the potential nucleic acid contamination associated with mNGS, together with its complicated procedures, the extended times required for obtaining reports, and its high cost also limit its use in clinical practice [35–37]. For now, we still hope to explore potentially valuable biomarkers using more extensive clinical tests. It should also be noted that the cytokine response to inflammation is nonspecific, and abnormal expression can also occur in other infections such as periodontitis, bacterial meningitis, and coronavirus disease 2019 (COVID-19) [38–40]. Therefore, the use of cytokines in CML pulmonary bacterial infection should be used in conjunction with other examinations and clinical symptoms.

## 5. Conclusions

TKI treatment did not affect cytokine expression in CML patients. However, the levels of Th1/2/17 cytokines were found to be significantly elevated in CML patients with pulmonary bacterial infection. In particular, the abnormal elevation of IL-6, IL-8, and IL-10 levels indicates the possibility of pulmonary bacterial infection in patients.

## Figures and Tables

**Table 1:** Basic characteristics of patients.

Parameters	No bacterial infection in the lungs (*n* = 172)	Bacterial lung infection (*n* = 65)	Bacterial and fungal infections of the lungs (*n* = 16)	HBV infection (*n* = 19)
Gender (male/female)	98/74	43/22	10/6	7/12
Age (years)	50 (36–59)	32 (25–44)	34 (23–44)	39 (28–52)
Temperature (°C)	36.5 (36.2–36.8)	36.8 (36.5–37.2)	37.0 (36.4–37.6)	36.5 (36.3–36.8)
PCT (ng/ml)	0.05 (0.03–0.13)	0.35 (0.10–0.55)	0.38 (0.20–3.12)	0.06 (0.03–0.37)
CRP (mg/l)	2.75 (1.83–5.21)	15.65 (8.17–54.85)	78.50 (13.75–113.50)	2.70 (1.69–19.98)

**Table 2:** Comparison of cytokine expression between uninfected patients receiving TKI treatment and non-TKI treated patients.

Parameters (pg/ml)	TKI treatment (*n* = 74)	No TKI treatment (*n* = 98)	*P*
IL-4	2.86 (1.77–4.05)	2.08 (1.26–2.79)	0.5500
IL-5	1.79 (1.08–2.56)	1.60 (1.01–2.30)	0.8370
IL-6	4.82 (2.54–8.54)	4.76 (2.54–7.25)	0.7110
IL-8	6.03 (3.37–11.67)	5.11 (2.85–8.27)	0.8740
IL-10	3.30 (2.22–4.73)	2.95 (1.92–4.05)	0.1720
IL-12p70	4.02 (2.45–4.78)	3.81 (2.90–4.86)	0.0510
IL-1*β*	1.76 (1.04–2.46)	1.64 (1.04–2.24)	0.7470
IL-2	2.81 (1.51–4.09)	2.48 (1.58–3.62)	0.2150
IFN-*γ*	2.03 (1.12–3.08)	1.88 (0.97–2.81)	0.1160
TNF-*α*	2.17 (1.18–2.93)	1.76 (1.03–2.74)	0.2610
TNF-*β*	2.07 (0.90–3.32)	1.93 (1.10–2.92)	0.1620
IL-17A	2.01 (1.39–3.02)	1.70 (1.04–2.48)	0.4360
IL-17F	2.28 (0.94–3.97)	2.23 (1.02–3.57)	0.0890
IL-22	1.19 (0.77–1.76)	1.19 (0.66–1.74)	0.1480

The difference in the expression of cytokines between TKI treatment and no treatment in noninfected patients was detected by the Mann–Whitney *U* test. *P* < 0.05 indicates a statistical difference.

**Table 3:** Expression of cytokines in different types of infection.

Parameter (pg/ml)	A: healthy group (*n* = 53)	B: no infection (*n* = 172)	C: bacterial lung infection (*n* = 65)	D: bacterial and fungal infections of the lungs (*n* = 16)	E: HBV infection (*n* = 19)	*P* (A vs. B)	*P* (B vs. C)	*P* (B vs. D)	*P* (B vs. E)	*P* (C vs. D)
IL-4	2.04 (1.20–2.64)	3.10 (1.77–4.60)	4.52 (2.28–7.43)	3.54 (2.07–6.52)	3.42 (2.08–4.20)	<0.0001	0.0051	0.2431	0.9129	0.6351
IL-5	2.92 (2.34-3.42	1.92 (1.16–2.45)	3.01 (1.89–3.52)	1.92 (1.42–3.61)	1.62 (1.09–2.82)	<0.0001	<0.0001	0.3489	0.7595	0.3077
IL-6	6.00 (4.87–7.27)	5.56 (3.03–7.75)	19.00 (8.16–64.29)	34.08 (3.94–111.36)	3.37 (1.80–11.93)	0.0563	<0.0001	0.0016	0.3103	0.9574
IL-8	5.81 (3.96–7.92)	6.15 (3.70–10.99)	20.36 (11.80–89.39)	28.57 (8.28–82.06)	7.83 (4.03–13.81)	0.2536	<0.0001	<0.0001	0.4085	0.9244
IL-10	3.61 (2.90–4.10)	3.70 (2.41–5.26)	5.53 (4.20–7.94)	6.07 (3.43–50.37)	3.91 (2.12–4.98)	0.7391	<0.0001	0.0011	0.8320	0.2402
IL-12p70	3.14 (2.87–3.38)	4.08 (2.93–5.04)	4.59 (3.33–5.67)	4.07 (3.64–5.81)	3.80 (1.73–5.16)	0.0000	0.0476	0.545	0.4160	0.7219
IL-1*β*	1.27 (0.93–1.65)	1.82 (1.19–2.35)	2.05 (1.58–3.03)	1.91 (1.22–2.58)	1.22 (0.97–2.59)	0.0003	0.0168	0.5989	0.2377	0.4302
IL-2	2.60 (1.93–3.18)	2.88 (1.78–4.52)	3.51 (2.52–6.01)	3.45 (2.24–6.70)	2.14 (0.75–3.51)	0.1201	0.0137	0.1710	0.0516	0.9433
IFN-*γ*	3.04 (2.01–3.46)	2.31 (1.35–3.44)	2.95 (2.09–4.22)	2.46 (1.84–3.41)	1.75 (1.47–3.85)	0.0121	0.0014	0.4408	0.9008	0.2086
TNF-*α*	3.66 (2.77–5.00)	2.36 (1.35–3.44)	3.28 (2.42–4.72)	3.28 (1.64–5.50)	2.59 (1.45–4.51)	<0.0001	<0.0001	0.0523	0.4337	0.9811
TNF-*β*	3.12 (2.77–3.60)	2.29 (1.25–3.33)	3.18 (2.10–4.06)	2.97 (1.95–3.73)	2.86 (0.98–3.38)	<0.0001	0.0004	0.0808	0.7628	0.6352
IL-17A	2.17 (1.56–3.46)	1.88 (1.28–2.87)	2.40 (1.21–4.41)	2.41 (1.49–3.83)	1.90 (1.06–4.82)	0.1170	0.0351	0.1914	0.5831	0.9716
IL-17F	4.30 (3.90–5.38)	2.76 (1.47–4.41)	3.73 (2.59–5.05)	3.47 (2.87–5.03)	1.86 (1.00–4.38)	<0.0001	0.0011	0.0728	0.2620	0.8125
IL-22	1.02 (0.73–1.41)	1.38 (0.77–2.16)	2.06 (1.25–3.21)	1.82 (1.07–2.31)	1.05 (0.45–1.62)	0.0110	0.0023	0.3006	0.1697	0.2857

The difference in the expression of cytokines between the patient's lungs with bacterial infection and without infection was detected by the Mann–Whitney *U* test. *P* < 0.05 indicates a statistical difference.

**Table 4:** Cut-off values of cytokines (AUC ≥ 0.70), CRP, PCT, and body temperature between uninfected patients and those with bacterial lung infection.

Parameters	AUC	Cut-off	*P*	Proportion of infections exceeding the cut-offvalue
CRP (mg/l)	0.80	6.18	<0.0001	64.86% (48/74)
PCT (ng/ml)	0.71	0.25	<0.0001	75.61% (31/41)
Body temperature (°C)	0.68	36.8	<0.0001	45.78% (38/83)
IL-5 (pg/ml)	0.73	2.41	<0.0001	48.31% (43/89)
IL-6 (pg/ml)	0.84	13.78	<0.0001	83.33% (45/54)
IL-8 (pg/ml)	0.82	14.35	<0.0001	67.16% (45/67)
IL-10 (pg/ml)	0.71	4.01	<0.0001	41.46% (51/123)
TNF-*α* (pg/ml)	0.74	3.00	<0.0001	43.33% (39/90)
IL-6, IL-8 and IL-10	—	At the same time ≥	—	93.55% (29/31)

Cut-off values of IL-5, IL-6, IL-8, IL-10, TNF-*α*, PCT, and CRP. Temperature are determined by the ROC curve. *P* < 0.05 indicates a statistical difference.

## Data Availability

The original data and supplementary graphics are supplemented in the supplementary documents section.

## Supplementary Materials

Supplementary Materials Supplementary Figure 1: the Mann–Whitney *U* test was used to examine the differences between patients with respiratory bacterial infection, patients with respiratory bacterial and fungal infections, patients without bacterial infection, and patients with HBV infection. (A) IL-4, (B) IL-12p70, (C) IL-1*β*, (D) IL-2, (E) IFN-*γ*, (F)TNF-*β*, (G) IL-17A, (H) IL-17F, and (I) IL-22. ^*∗*^*P* < 0.05,  ^*∗∗*^*P* < 0.05,  ^*∗∗∗*^*P* < 0.001,  ^*∗∗∗∗*^*P* < 0.0001, ns: no statistical difference.
