# Supplementary figures and images for: Expression of Th1/2/17 Cytokines in CML with or without Pulmonary Bacterial and Fungal Coinfection
Source: J Oncol. 2023 Apr 18;2023:6318548. doi: 10.1155/2023/6318548 (PMC10129429; doi:10.1155/2023/6318548)

## Slide 1
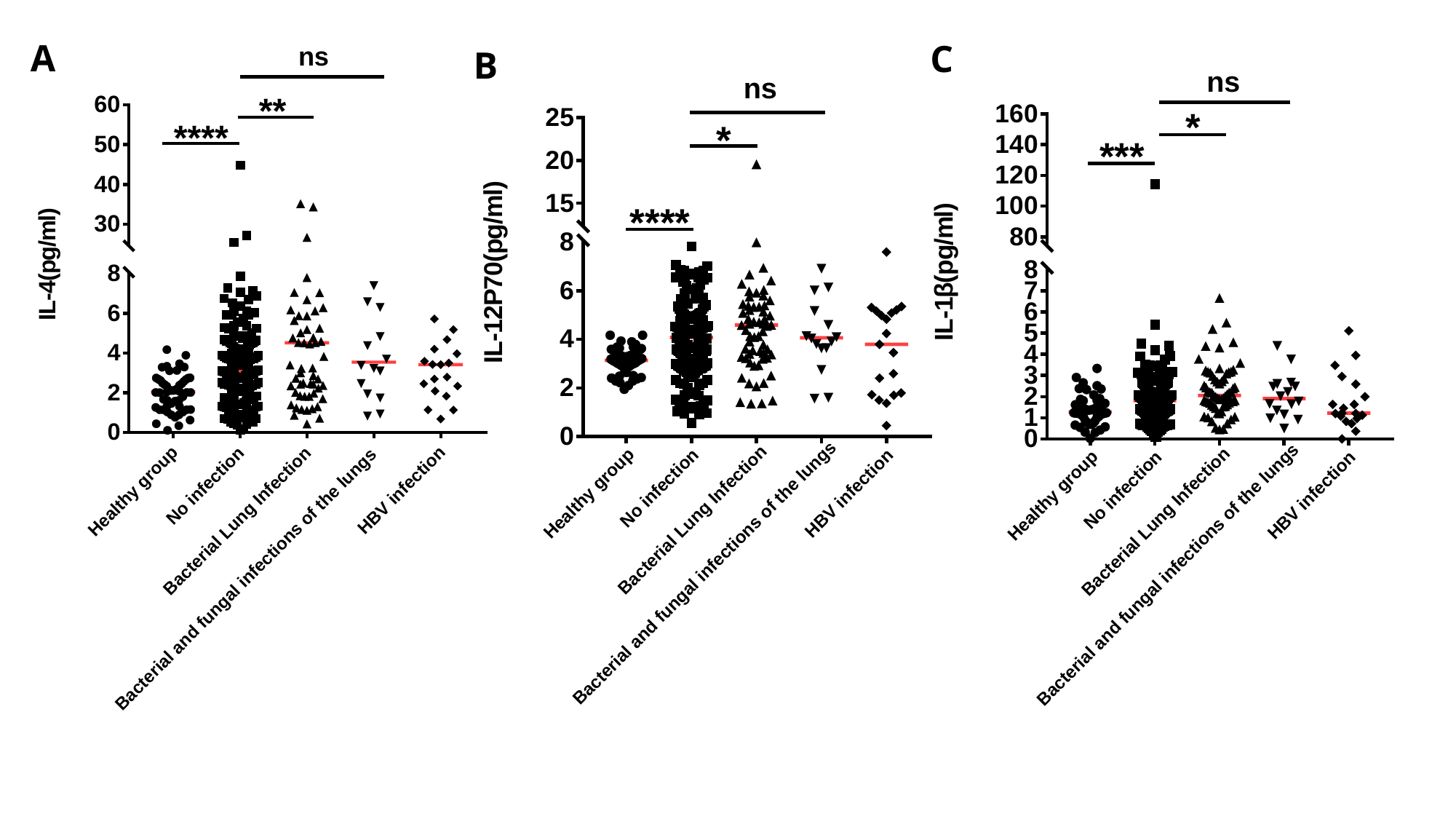

A
C
B

## Slide 2
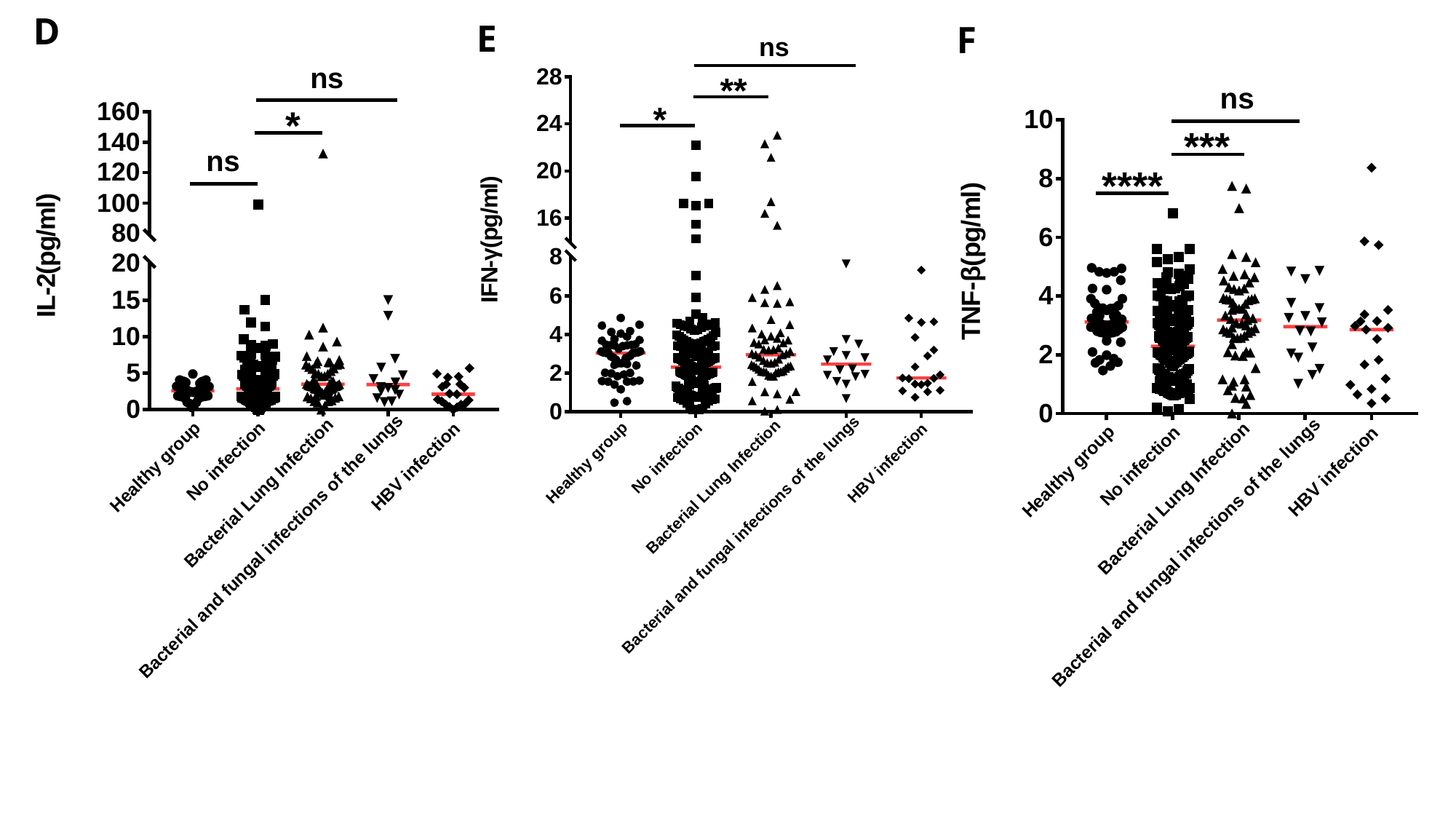

D
E
F

## Slide 3
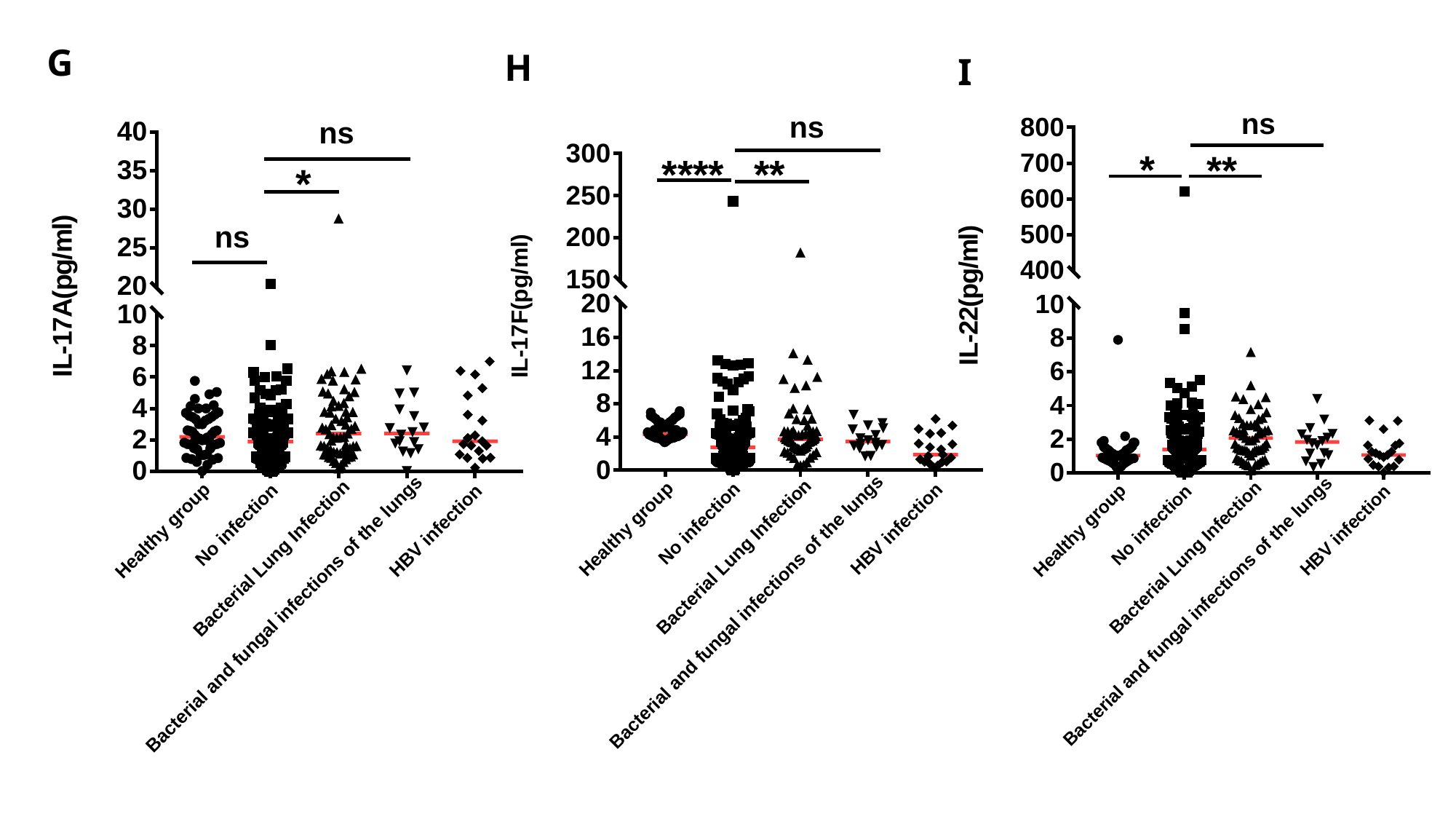

G
H
I

Supplement: Supplementary Materials — Supplementary Figure 1: the Mann–Whitney U test was used to examine the differences between patients with respiratory bacterial infection, patients with respiratory bacterial and fungal infections, patients without bacterial infection, and patients with HBV infection. (A) IL-4, (B) IL-12p70, (C) IL-1β, (D) IL-2, (E) IFN-γ, (F)TNF-β, (G) IL-17A, (H) IL-17F, and (I) IL-22. ∗P < 0.05, ∗∗P < 0.05, ∗∗∗P < 0.001, ∗∗∗∗P < 0.0001, ns: no statistical difference. [file 6318548.f1.pptx]
